# Maternal transmission gives way to social transmission during gut microbiota assembly in wild mice

**DOI:** 10.1186/s42523-023-00247-7

**Published:** 2023-05-31

**Authors:** Klara M. Wanelik, Aura Raulo, Tanya Troitsky, Arild Husby, Sarah C. L. Knowles

**Affiliations:** 1 Department of Biology, University of Oxford, Oxford, UK; 2 Department of Computing, University of Turku, Turku, Finland; 3 Evolutionary Biology Centre, Department of Ecology and Genetics, Uppsala University, Uppsala, Sweden

**Keywords:** Gut microbiome, Mice, Transmission, *Muribaculaceae*, Maternal transmission, Rodent, *Apodemus*, 16S.

## Abstract

**Background:**

The mammalian gut microbiota influences a wide array of phenotypes which are relevant to fitness, yet knowledge about the transmission routes by which gut microbes colonise hosts in natural populations remains limited. Here, we use an intensively studied wild population of wood mice (*Apodemus sylvaticus*) to examine how vertical (maternal) and horizontal (social) transmission routes influence gut microbiota composition throughout life.

**Results:**

We identify independent signals of maternal transmission (sharing of taxa between a mother and her offspring) and social transmission (sharing of taxa predicted by the social network), whose relative magnitudes shift as hosts age. In early life, gut microbiota composition is predicted by both maternal and social relationships, but by adulthood the impact of maternal transmission becomes undetectable, leaving only a signal of social transmission. By exploring which taxa drive the maternal transmission signal, we identify a candidate maternally-transmitted bacterial family in wood mice, the *Muribaculaceae*.

**Conclusion:**

Overall, our findings point to an ontogenetically shifting transmission landscape in wild mice, with a mother’s influence on microbiota composition waning as offspring age, while the relative impact of social contacts grows.

**Supplementary Information:**

The online version contains supplementary material available at 10.1186/s42523-023-00247-7.

## Background

The gut microbiota has important effects on host phenotypes, from immune development [1], to pathogen resistance [2] and behaviour [3]. An individual’s microbiota is shaped by a variety of forces, including its own genotype [4, 5] and environmental factors like diet [6, 7]. However, microbial transmission processes also play a critical role in shaping these communities, by fundamentally determining the pool of microbes that arrive at host epithelial surfaces. Gut microbe transmission can occur at various stages throughout an animal’s life, with colonising microbes deriving from various sources, including other conspecifics and the environment.

In many species, the mother constitutes an important initial source of microbes that can colonise the gut before, during or after birth. In mammals, although the existence of *in utero* gut microbe transmission is contentious [8], live birth and maternal care provide ample opportunity for vertical transmission during and after birth. Maternal transmission of gut microbes has been well described in humans and inbred laboratory mice, and microbes from the mother’s vaginal, oral, skin, milk and gut microbiota can all be transferred to offspring [9–12]. The vagina constitutes a key source of microbes that can colonise infants during birth [11–15]. In neonates of both mice and humans, the gut microbiota most closely resembles the maternal vaginal microbiota [11, 13, 16]. The maternal gut is also a key source of gut colonists for young mammals. Well-resolved strain-tracking data from humans indicates that vaginally-derived bacterial strains are transient colonisers and that, among body sites, the maternal gut is the source of the majority of strains transmitted from mother to infant in the first four months of life, including more persistent colonisers of the infant gut [9]. Maternal gut microbes may be transferred through exposure to faeces during birth, through breast milk (as microbes found in breast milk likely originate from the gut [17, 18]), via coprophagy [19], or indirectly through nesting material [20]. Although these pathways have been relatively well-studied in humans and laboratory mice, far less is understood about the relative significance, pathways and timing of maternal transmission in wild animals.

Social transmission of microbes from other conspecifics constitutes another important source of transmission that can shape the gut microbiota [21]. Microbes can be shared via direct social interactions such as grooming, aggression or mating, through coprophagy, or via indirect transmission through shared use of space, such as use of the same nest. A growing body of evidence now illustrates the significant influence social transmission can have on the mammalian gut microbiota. For example, in primates, mice and horses, research shows that individuals from the same social group frequently share more microbial taxa and, within social groups or populations, the strength of social interactions predicts microbiota similarity [22–25].

These two major routes of acquiring gut microbes from conspecifics—maternal and social—may each be expected to vary in importance as young animals mature and age. While maternal transmission should be strongest when offspring are interacting closely with their mother (typically in early life), social transmission can take place whenever social interactions are occuring, which for many species constitutes a longer part of the life-course. In mammals, one may therefore expect maternal transmission to predominate early in life, with social transmission beginning later and increasing as offspring mature and engage in more, and perhaps different forms of social interaction. However, currently there is only limited data from natural systems to explore this. One human study found no evidence that the impact of social microbial transmission changed with age [26], but studies in wild animals dissecting the relative influence of maternal and social transmission, and how these change with age, are currently lacking.

Accurately detecting and isolating distinct influences of maternal and social transmission on the gut microbiome in free-living animals is challenging. Maternal transmission, for example, is commonly detected by comparing the bacterial taxa present in a mother and her offspring, with a signal of such transmission occurring when mother-offspring pairs share more bacterial taxa than otherwise comparable but genetically unrelated pairs. However, a mother and her offspring may share bacterial taxa for various reasons besides maternal transmission. These include acquisition through exposure to a shared environmental reservoir or other social contact, or similar (within-host) selection processes that retain or promote the same bacteria, for example through shared genetics or a similar diet. Therefore, in order to isolate a true signal of maternal transmission these other confounding variables need to be accounted for. Studies attempting to isolate the effect of maternal transmission in wild systems are rare (but see [27–29]), and the signal of maternal transmission is not always robustly isolated from other confounding variables. A second challenge is that the influence of maternal transmission may vary with offspring age. This has been shown in humans and laboratory mice, where the signal of maternal transmission is strongest after birth and gradually decreases over time [30–32]. In wild systems, a signal of maternal transmission could change with offspring age for several non-mutually-exclusive reasons. First, the type, frequency or duration of interaction between a mother and her offspring may change over time, leading to a change in the level of microbial transmission between mother and offspring. For example, a mother may nurse her offspring less frequently as they approach weaning, providing less opportunity for microbial transmission. Second, microbial taxa that are maternally transmitted early in life may be outcompeted and replaced by those that are acquired later in life from other sources, for example via social transmission or from the environment. Finally, the infant gut microbiota may go through a process of succession that begins with colonisation by maternal (pioneer) microbes, but then may become increasingly shaped by other ecological processes such as microbial selection through diet, microbe-microbe interactions and host immunity, making it more difficult to detect the signal of early-life maternal transmission.

Here, we use a wild population of wood mice (*Apodemus sylvaticus*) to disentangle the distinct influences of maternal and social transmission on the gut microbiota, and assess how their relative influences change throughout life. We build on our previous work in which we detected a clear signal of social transmission in this population (sharing of taxa being predicted by the social network [23]). Wood mice are well-suited for disentangling the influences of maternal and social transmission, as they are a non-group-living species in which offspring become independent from their mothers after weaning when they emerge from their nest and disperse [33, 34]. This life-history means social networks are independent of genetic relatedness in this species [23], unlike in many group-living mammals. We use a longitudinal set of faecal samples from a wild population of wood mice for which we also have a rich set of metadata on other covariates that could influence mother-offspring microbiota similarity, including social relationships, spatial locations and seasonality. Using a Bayesian dyadic mixed-effects modelling approach, we control for these potential confounders to isolate the effect of maternal transmission and examine how its strength, relative to that of social transmission, changes with age.

## Methods

Data were collected from November 2014 to December 2015 from a wild population of wood mice in a 2.47 ha mixed woodland plot (Nash’s Copse) at Imperial College’s Silwood Park campus, UK. Field methods are described in detail in [23]. Briefly, sterilised live traps were set for one night every 2–4 weeks in an alternating checkerboard design, to ensure even spatial coverage over time. All traps contained bedding and a standardised bait of 8 peanuts and a slice of apple. At first capture, all mice were injected subcutaneously with a passive integrated transponder tag (PIT-tag) for permanent identification, and a small ear snip was taken for genotyping. At each trapping, demographic data on captured animals was recorded (e.g. sex and age), and faecal samples were collected from traps for gut microbiota analysis and stored at − 80^o^C within 8 h of collection.

### Social network and space use

Data on mouse space use and the social network was collected in parallel to trapping using a set of nine custom-built PIT-tag loggers that were regularly rotated around the trapping grid to achieve even coverage, as described in [23]. Individuals were considered socially associated with each other if they were detected at the same location on the same night (12-hour period, 6pm to 6am). These data were used to calculate an adjusted version of the Simple Ratio Index (“Adjusted SRI”, see Supplementary information in [23]), that accounts for variable overlap in individual lifespans (i.e. time between first and last logger observation).

### Aging

Individuals were aged as either juvenile, subadult or adult based on pelage and weight (body mass range: juvenile = 10.4–14.5 g; subadult = 13.0–21.5 g; adult = 15.1–40.4 g). Most samples were from adults (*n* = 152 samples) and as we had limited samples from juveniles (*n* = 7 samples) we grouped these with samples from sub-adults (*n* = 65 samples) to create one immature age class (*n* = 72 samples).

### Kinship analysis

To derive estimates of host genetic relatedness, ear tissue samples were used to genotype mice at eleven microsatellite loci. A pedigree was then reconstructed using COLONY 2.0.6.5 [35], a program for parental and sibship inference from genotype data. The resulting pedigree was checked against trapping data to remove impossible relationships based on age and trapping date. Finally, kinship results were transformed into genetic relatedness values (unrelated = 0; parent-offspring pair = 0.5; sibling = 0.5; half-sibling = 0.25). Full details of genotyping methods including the target regions chosen and pedigree reconstruction methods are provided in [23].

### Gut microbiota characterisation

The gut microbiota was characterised for 224 faecal samples belonging to 70 genotyped wood mice, including 22 samples from 6 mothers and 37 samples from 17 of their offspring. Microbiota methods for this dataset have been described previously in [23]. Briefly, microbiota profiling involved amplicon sequencing of the 16S rRNA gene (V4 region), with sequence data processed through the DADA2 pipeline v1.6.0 [36] to infer amplicon sequence variants (ASVs) and taxonomy assigned using the GreenGenes Database (Consortium 13.8). Using the package *phyloseq* [37], ASV counts were normalised to proportional abundance within each sample [38] and singleton ASVs were removed as well as those belonging to non-gut microbial taxa (Cyanobacteria, Mitochondria).

###  Statistical analyses

All analyses were conducted in R version 4.0.2 [39]. The samples characterised involved 4,167 unique pairs of individuals (of which 15 were mother-offspring pairs), and 24,765 (between-individual) sample-pairs (of which 182 were mother-offspring sample-pairs). To describe compositional microbiota variation, package *vegan* [40] was used to calculate Jaccard distances and Bray-Curtis dissimilarities among samples. We used the Jaccard Index (the proportion of ASVs shared between a pair of samples, 1 − Jaccard distance) as our primary measure of microbiota similarity, as we considered this metric most relevant for investigating microbial transmission among hosts. However, we also tested whether results were robust to the type of distance metric used, by repeating key analyses using Bray-Curtis similarity (which measures similarities in ASV relative abundance as well as presence-absence, 1 − Bray-Curtis dissimilarity; presented in Additional file 2: Table S2).

#### Associations between mother-offspring status and microbiota similarity

We used Bayesian (dyadic) regression models in package *brms* [41, 42] to model the impact of multiple predictors on pairwise microbiota similarity (Jaccard Index) with a Beta family and logit link. All types of kinship pair were included in our analysis (unrelated pairs, father-offspring pairs, mother-offspring pairs, sibling pairs and half-sibling pairs) and the binary predictor mother-offspring status (that captured whether a pair of individuals were a mother-offspring pair, 1, or not, 0), tested for an effect of maternal transmission. Since individual mice were often sampled multiple times, and all individuals were included in multiple pairwise similarity measures, we included two multi-membership random effects to account for the non-independence inherent to this type of data: (1) a random intercept term for the individuals in each dyad (Individual A + Individual B) and (2) a random intercept term for the samples in each dyad (Sample A + Sample B). As in [23], we included other variables that were either suspected or previously demonstrated drivers of dyadic microbiota similarity. Since we used a dyadic framework, these predictor variables were coded as similarities, distances or associations describing a *pair* of samples. These included genetic relatedness (unrelated pair = 0; parent-offspring pair = 0.5; sibling pair = 0.5; half-sibling pair = 0.25), social association (adjusted SRI), age class similarity (same age class = 1; different age class = 0), sex similarity (same sex = 1; different sex = 0), spatial distance (distance between individuals’ mean spatial coordinates from logger records) and sampling interval (time in days between which samples were taken). We excluded all pairs of samples where both individuals were immature (immature-immature pairs; *n* = 2,566 sample-pairs), leaving only adult-immature and adult-adult pairs. To allow us to test whether the mother-offspring effect (signal of maternal transmission) varied with offspring age class, we also excluded a small number of mother-offspring sample-pairs where the mother was sampled as an immature (*n* = 16 sample-pairs). This meant that age class similarity among mother-offspring pairs was a function of offspring age class only i.e. mother-offspring pairs could only differ in age class (adult-immature pair) if offspring were immature, vs. having the same age class (adult-adult pair) if both mother and offspring were adult. We then included an interaction term between the variable mother-offspring status and age class similarity in order to test whether the maternal transmission effect varied with offspring age. In the end, 22,199 sample-pairs were included in the analysis, of which 170 were mother-offspring. All models were checked for convergence by visual inspection of trace plots and the *Rhat* statistic.

#### Identifying which bacterial taxa associate with mother-offspring status

We used the same approach used to identify candidate socially-transmitted bacterial taxa in [23], to here identify candidate maternally-transmitted bacterial taxa. We tested how each bacterial family influenced the effect size of (1) the main effect of mother-offspring status, and (2) the interaction between mother-offspring status and age class similarity. We recalculated the Jaccard Index excluding each bacterial family in turn, then compared effect sizes and credible intervals from the same models run in the package *MCMCglmm* [43] using these indices.

## Results

### Identifying maternal and social transmission signals

We found no association between mother-offspring status and social association (Mantel test: *r* = 0.01, *p* = 0.17), allowing us to dissect the distinct influences of maternal and social transmission on the gut microbiota. Across the whole dataset, we identified a clear signal of maternal transmission in wild mice, since mother-offspring status positively predicted the proportion of shared gut microbial ASVs (posterior mean = 0.09, 95% CI = 0.05, 0.13; Additional file 1: Table S1), when controlling for a range of confounding variables that could also influence mother-offspring microbiota similarity (social association, spatial distance, sampling interval and genetic relatedness). This maternal transmission effect was weaker overall than the social transmission effect (posterior mean = 0.38, 95% CI = 0.34, 0.42; Additional file 1: Table S1). Consistent with [23], we found that other variables also predicted microbiota similarity, including spatial distance and sampling interval (Additional file 1: Table S1). Consistent results were obtained when using Bray-Curtis similarities (Additional file 2: Table S2).

### The influence of age class on maternal and social transmission signals

**Fig. 1:**
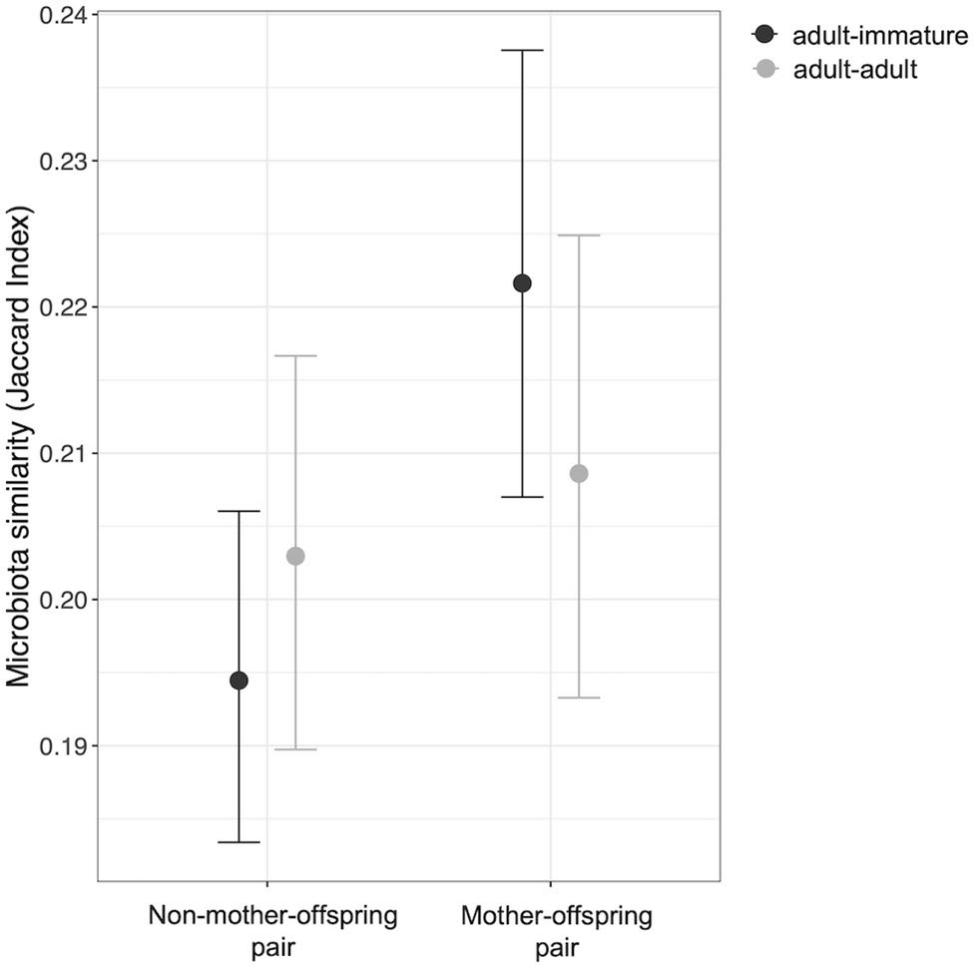
**The maternal transmission signal depends on age class similarity (adult-immature or adult-adult pair).** Estimates (points) and their 95% credible intervals are plotted from Bayesian regression (*brms*) models controlling for other confounding variables, with pairwise microbiota similarity among hosts (Jaccard Index) as the response. Among mother-offspring pairs where one is an adult and the other immature (adult-immature pairs), the immature individual is always the offspring. Non-mother-offspring pairs include all other types of kinship pairs (unrelated pairs, father-offspring pairs, sibling pairs and half-sibling pairs)

**Fig. 2:**
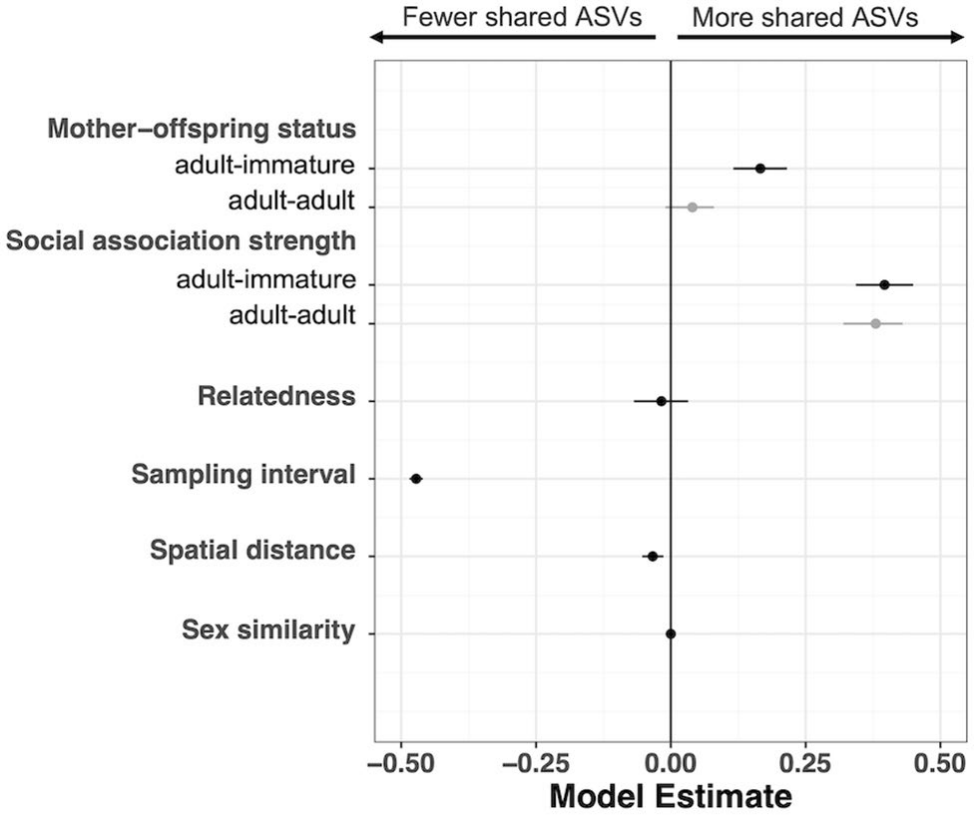
**Both maternal and social relationships predict gut microbiota similarity early in life (adult-immature pairs), with the the magnitude of the maternal effect waning in adulthood (adult-adult pairs).** Effect size estimates (points) and their 95% credible intervals are plotted from Bayesian regression (*brms*) models with pairwise microbiota similarity among hosts (Jaccard Index) as the response. Where credible intervals do not overlap zero, a variable significantly predicts microbiota similarity

When an interaction term between mother-offspring status and age class similarity was included in the model, this interaction term was significant (posterior mean = − 0.13, 95% CI = − 0.19, − 0.07; Additional file 1: Table S1), indicating that the mother-offspring effect was stronger when offspring were immature. Among adult-immature pairs, mother-offspring pairs shared a higher proportion of ASVs than non-mother-offspring pairs (posterior mean for mother-offspring status = 0.17, 95% CI = 0.12, 0.22; Additional file 1: Table S1), whereas among adult-adult pairs, mother-offspring and non-mother-offspring pairs shared a similar proportion of ASVs (posterior mean for mother-offspring status = 0.04, 95% CI = − 0.01, 0.08; Additional file 1: Table S1; Fig. 1). We also included an interaction term between social-association strength and age class similarity, but this was not significant suggesting that the signal of social transmission remained consistent throughout life (posterior mean for interaction term = − 0.02, 95% CI = − 0.09, 0.05; Additional file 1: Table S1). Although we found that the maternal transmission effect was markedly weaker than the social transmission effect overall (see above), this difference was clearer when separating by age class; during early life (adult-immature pairs), both social and maternal transmission signals were clearly detectable, with the social signal approximately twice as strong as the maternal signal (Fig. 2). However, by adulthood (adult-adult pairs) the maternal signal had reduced to an undetectable level, leaving only a strong and consistent signal of social transmission (Fig. 2). Again, consistent results were obtained when using Bray-Curtis similarities (Additional file 2: Table S2).

### Identifying candidate maternally-transmitted bacterial taxa

**Fig. 3:**
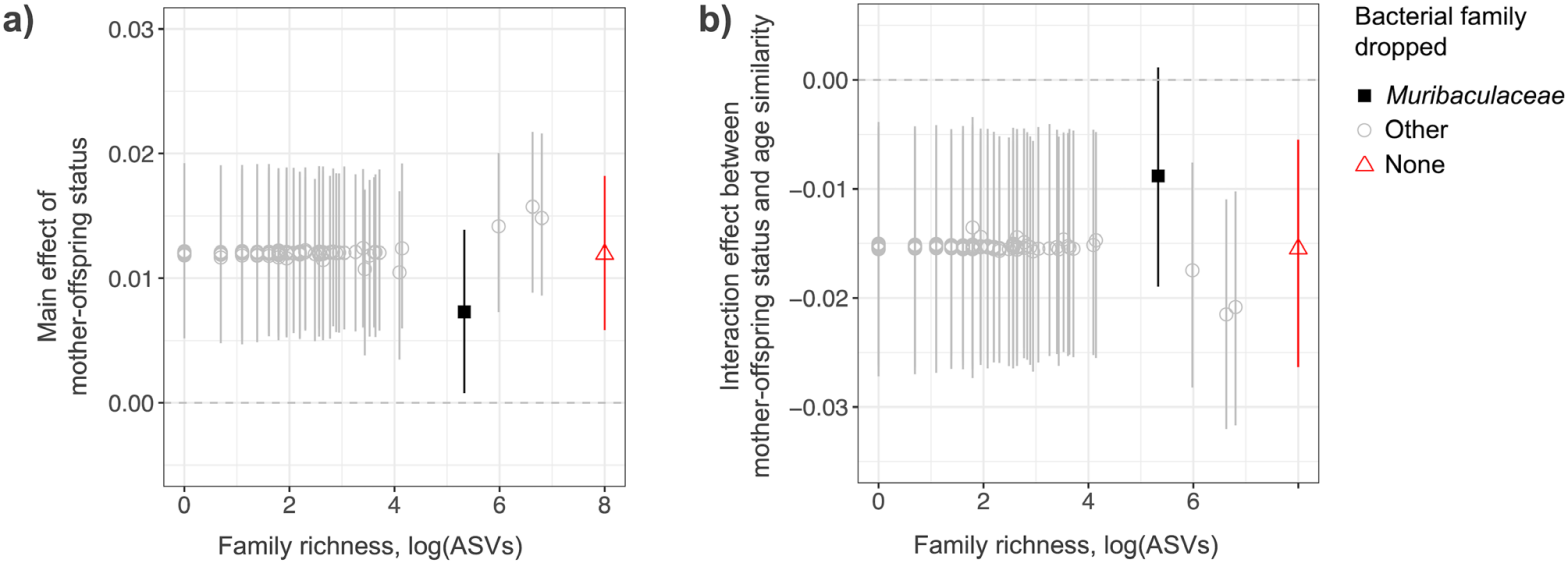
**The influence of specific bacterial families on the maternal transmission signal.** Effect sizes for (a) the main effect of mother-offspring status, and (b) the interaction between mother-offspring status and age class similarity and 95% credible intervals are plotted from 146 Bayesian regression models, in each of which a single bacterial family was excluded. One might expect to see a relationship between the species richness of a dropped family and the resulting effect size—with the exclusion of a more diverse family being associated with a smaller resulting effect size. To assess this, effects are plotted against the species richness of each dropped family (logged number of ASVs), though no such relationship is observed. Effect sizes and their 95% credible intervals from the full model (with no bacterial families dropped) are included for reference on the far right (in red)

The main effect of mother-offspring status remained significant (95% credible intervals did not include zero) in all models where a single bacterial family was excluded, suggesting that it did not depend entirely on any single bacterial family (Fig. 3a; Additional file 3: Table S3). However, excluding the *Muribaculaceae* did weaken the main effect size considerably (taking the posterior mean from 0.012 to 0.007; Fig. 3a; Additional file 3: Tables S3). The interaction effect between mother-offspring status and age class similarity showed broadly the same pattern—with the exclusion of the *Muribaculaceae* weakening the interaction effect size considerably (taking the posterior mean from − 0.015 to − 0.009; Fig. 3b; Additional file 4: Table S4). However, the interaction effect did not remain significant (95% credible interval did include zero) when the *Muribaculaceae* were excluded, suggesting that it depended on this bacterial family. The disproportionate influence of the *Muribaculaceae* compared to other bacterial families on these maternal transmission signals was not related to its ASV diversity (and therefore the number of ASVs lost from beta diversity metrics when it was excluded; Fig. 3).

## Discussion

In this study, we examine the relative influence of maternal and social transmission on the gut microbiota as hosts age in a wild mouse population. We do this by drawing on a rare example of a longitudinal dataset of paired mother-offspring faecal samples taken from the wild, for which we also have a rich set of metadata. By controlling for other confounding variables we are able to isolate distinct maternal and social transmission signals. We show that which individuals are important for shaping an offspring’s gut microbiota composition changes through life—while the mother’s importance declines, the importance of other social contacts remains stable from the early independent phase into adulthood. Our finding of a maternal gut microbial transmission signal parallels those observed in inbred laboratory mice [10, 11] and suggests this transmission pathway still has a notable influence in the wild, remaining detectable post-weaning despite the many other processes at play.

Our estimate of the maternal transmission signal is likely conservative as all the offspring we sampled as part of this study were weaned (and trappable). Despite this, we still found a maternal transmission signal, which may be stronger at or shortly after birth, had we been able to sample at this point. We are unable to determine from our data how much influence social transmission might have had at the very earliest stage of life (pre-weaning), but we expect this to be minimal since before weaning wood mice are raised solely by their mother in underground burrows, and mothers are territorial during this time [44]. What we can say, is that by the time individuals are independent and capable of being caught in live traps, social transmission already has a clear influence on gut microbiota composition.

Of course, some forms of maternal transmission may be considered somewhat ‘social’ in nature—driven by close proximity between a mother and her offspring within the nest. A close link between maternal and social transmission is consistent with our finding that the same bacterial family (the *Muribaculaceae)* previously shown to influence the social transmission signal in this system [23] also had a disproportionate influence on the maternal transmission signal here [23]. The *Muribaculaceae* are non-spore forming and anaerobic [45], which means they cannot persist for any length of time in the environment. These life history traits are consistent with this taxon being transmitted between individuals, either from a mother to her offspring during early-life interactions underground, or from one conspecific to another through social interaction. Studies in other mammals have identified other maternally-transmitted taxa which were not detected as being maternally transmitted here. In humans, it is well established that bifidobacteria are maternally transmitted [12, 46–49]. More recently, bifidobacteria have been shown to be commonly maternally transmitted across a wide range of mammals (including primates and non-primates) [46]. Although bifidobacteria were not identified as important for maternal transmission in this study, they were detected in the wood mouse gut microbiota. Other candidate maternally-transmitted taxa include *Lactobacillus* [12, 50] and *Bacteroides* [11, 28]. All these taxa are implicated in the degradation of milk oligosaccharides. Gut-associated *Lactobacillus*, for example, are found in human breastmilk, can be transmitted to neonates via breastfeeding [18] and are involved in milk breakdown [15]. We might then expect such taxa to be transitional, giving way to other taxa (e.g. fibre fermenters) as diet changes in later life, as appears to occur for *Lactobacillus* in humans [50]. More data, including higher resolution microbiome profiling of offspring and mothers in early life, would be needed to confirm which taxa are maternally transmitted in wood mice, the duration of their colonisation, and functional significance.

Transmission events early in life may have long-lasting effects on an individual. In terms of shared taxa, we found that adult offspring were no more similar to their mother than to any other individual in the population. However, maternal impacts on an adult’s microbiota that are harder to detect may nonetheless persist, if maternal transmission events early in life give rise to predictable patterns of microbial succession that permanently change a microbial community. Our analyses would not detect such a change, but higher resolution time-series analyses combined with AI could potentially be used to test this hypothesis in the future. Even transient changes in microbiota early in life can shape development in key ways. For example, a recent study suggested that such transient changes may help establish alternative development trajectories in industrialised and non-industrialised human populations [51]. Short-term antibiotic-driven microbiota perturbation in early life has also been linked to long-term host metabolic effects implicated in obesity [52], and long-term immunological effects implicated in asthma and autoimmune diseases [53]. Future wild studies that assess links between early-life microbial communities and later-life traits associated with health or fitness (similar to [54]) would be useful in this respect. We suggest wild mouse systems, like that used here, could provide a tractable system in which to quantify these long-term, and potentially profound, effects of maternal transmission.

## Electronic supplementary material

Below is the link to the electronic supplementary material.

**Additional file 1: Table S1.** Results of *brms* models testing the effect of mother-offspring status and covariates on microbiota similarity (Jaccard Index). Significant terms (where 95% credible intervals do not include zero) are shown in bold. Est. Error indicates the standard deviation of the posterior distribution

**Additional file 2: Table S2.** Results of *brms* models testing the effect of mother-offspring status and covariates on microbiota similarity (Bray-Curtis). Significant terms (where 95% credible intervals do not include zero) are shown in bold. Est. Error indicates the standard deviation of the posterior distribution

**Additional file 3: Table S3**. Results of 146 *brms* models testing the main effect of mother-offspring status, in each of which a single bacterial family was dropped. For each dropped family, we include the species richness of the dropped family, the effect size and its 95% credible interval after the family was dropped, as well as the change in the effect size after the family was dropped (compared to the effect size from the full model; any change indicated in bold; a negative change indicating a decrease in effect size and vice versa). Families are ranked on species richness (logged number of ASVs)

**Additional file 4: Table S4**. Results of 146 *brms* models testing the interaction between mother-offspring status and age class, in each of which a single bacterial family was dropped. For each dropped family, we include the species richness of the dropped family, the effect size and its 95% credible interval after the family was dropped, as well as the change in the effect size after the family was dropped (compared to the effect size from the full model; any change indicated in bold; a negative change indicating a decrease in effect size and vice versa). Families are ranked on species richness (logged number of ASVs)

## Data Availability

The dataset supporting the conclusions of this article is available in the European Nucleotide Archive (ENA) under Accession no. PRJEB49639 (https://www.ebi.ac.uk/ena/browser/view/PRJEB49639).
