# Supplementary material for: Maternal transmission gives way to social transmission during gut microbiota assembly in wild mice
Source: Anim Microbiome. 2023 May 31;5:29. doi: 10.1186/s42523-023-00247-7 (PMC10230743; doi:10.1186/s42523-023-00247-7)
Supplement: Supplementary file 1 — Additional file 1: Table S1. Results of brms models testing the effect of mother-offspring status and covariates on microbiota similarity (Jaccard Index). Significant terms (where 95% credible intervals do not include zero) are shown in bold. Est. Error indicates the standard deviation of the posterior distribution [file 42523_2023_247_MOESM1_ESM.docx]

**Table S1** Results of *brms* models testing the effect of mother-offspring status and covariates on microbiota similarity (Jaccard Index). Significant terms (where 95% credible intervals do not include zero) are shown in bold. Est. Error indicates the standard deviation of the posterior distribution.

| **Without interaction terms** | | | | |
| --- | --- | --- | --- | --- |
|  | **Estimate** | **Est. Error** | **l-95% CI** | **u-95% CI** |
| Intercept | −1.25 | 0.04 | −1.32 | −1.17 |
| Sex similarity | 0.00 | 0.00 | −0.00 | 0.01 |
| **Spatial distance** | **−0.04** | **0.01** | **−0.05** | **−0.02** |
| **Temporal distance** | −**0.47** | **0.01** | **−0.49** | **−0.46** |
| Relatedness | −0.02 | 0.03 | −0.07 | 0.04 |
| **Social association strength** | **0.38** | **0.02** | **0.34** | **0.42** |
| **Mother-offspring status** | **0.09** | **0.02** | **0.05** | **0.13** |
| Age class similarity | 0.05 | 0.03 | −0.00 | 0.11 |
| **With interaction terms** | | | | |
|  | **Estimate** | **Est. Error** | **l-95% CI** | **u-95% CI** |
| Intercept | −1.25 | 0.04 | −1.32 | −1.17 |
| Sex similarity | −0.00 | 0.00 | −0.01 | 0.01 |
| **Spatial distance** | −**0.03** | **0.01** | **−0.05** | **−0.01** |
| **Temporal distance** | **−0.47** | **0.01** | **−0.48** | **−0.46** |
| Relatedness | −0.02 | 0.03 | −0.07 | 0.03 |
| **Social association strength** | **0.40** | **0.03** | **0.34** | **0.45** |
| Mother-offspring status | 0.17 | 0.03 | 0.12 | 0.22 |
| Age class similarity | 0.05 | 0.03 | −0.00 | 0.11 |
| Social association strength:Age class similarity | −0.02 | 0.04 | −0.09 | 0.05 |
| **Mother-offspring status:Age class similarity** | **−0.13** | **0.03** | **−0.19** | **−0.07** |
