# Supplementary material for: Maternal transmission gives way to social transmission during gut microbiota assembly in wild mice
Source: Anim Microbiome. 2023 May 31;5:29. doi: 10.1186/s42523-023-00247-7 (PMC10230743; doi:10.1186/s42523-023-00247-7)
Supplement: Supplementary file 2 — Additional file 2: Table S2. Results of brms models testing the effect of mother-offspring status and covariates on microbiota similarity (Bray-Curtis). Significant terms (where 95% credible intervals do not include zero) are shown in bold. Est. Error indicates the standard deviation of the posterior distribution [file 42523_2023_247_MOESM2_ESM.docx]

**Table S2** Results of *brms* models testing the effect of mother-offspring status and covariates on microbiota similarity (Bray-Curtis). Significant terms (where 95% credible intervals do not include zero) are shown in bold. Est. Error indicates the standard deviation of the posterior distribution.

| **Without interaction terms** | | | | |
| --- | --- | --- | --- | --- |
|  | **Estimate** | **Est. Error** | **l-95% CI** | **u-95% CI** |
| Intercept | −0.79 | 0.06 | −0.91 | −0.68 |
| Sex similarity | 0.00 | 0.01 | −0.02 | 0.01 |
| **Spatial distance** | **−0.10** | **0.02** | **−0.14** | **−0.06** |
| **Temporal distance** | **−0.46** | **0.01** | **−0.49** | **−0.43** |
| **Relatedness** | **−0.17** | **0.06** | **−0.28** | **−0.06** |
| **Social association strength** | **0.35** | **0.05** | **0.26** | **0.44** |
| **Mother-offspring status** | **0.22** | **0.04** | **0.13** | **0.31** |
| Age class similarity | 0.05 | 0.05 | −0.04 | 0.15 |
| **With interaction terms** | | | | |
|  | **Estimate** | **Est. Error** | **l-95% CI** | **u-95% CI** |
| Intercept | −0.79 | 0.06 | −0.91 | −0.68 |
| Sex similarity | 0.00 | 0.01 | −0.02 | 0.01 |
| **Spatial distance** | **−0.09** | **0.02** | **−0.14** | **−0.05** |
| **Temporal distance** | **−0.46** | **0.01** | **−0.49** | **−0.43** |
| **Relatedness** | **−0.17** | **0.06** | **−0.28** | **−0.06** |
| **Social association strength** | **0.32** | **0.06** | **0.20** | **0.45** |
| Mother-offspring status | 0.36 | 0.06 | 0.25 | 0.48 |
| Age class similarity | 0.06 | 0.05 | −0.04 | 0.15 |
| Social association strength:Age class similarity | 0.06 | 0.08 | −0.10 | 0.22 |
| **Mother-offspring status:Age class similarity** | **−0.27** | **0.07** | **−0.40** | **−0.13** |
