# Supplementary material for: Maternal transmission gives way to social transmission during gut microbiota assembly in wild mice
Source: Anim Microbiome. 2023 May 31;5:29. doi: 10.1186/s42523-023-00247-7 (PMC10230743; doi:10.1186/s42523-023-00247-7)
Supplement: Supplementary file 3 — Additional file 3: Table S3. Results of 146 brms models testing the main effect of mother-offspring status, in each of which a single bacterial family was dropped. For each dropped family, we include the species richness of the dropped family, the effect size and its 95% credible interval after the family was dropped, as well as the change in the effect size after the family was dropped (compared to the effect size from the full model; any change indicated in bold; a negative change indicating a decrease in effect size and vice versa). Families are ranked on species richness (logged number of ASVs) [file 42523_2023_247_MOESM3_ESM.docx]

**Table S3** Results of 146 *brms* models testing the main effect of mother-offspring status, in each of which a single bacterial family was dropped. For each dropped family, we include the species richness of the dropped family, the effect size and its 95% credible interval after the family was dropped, as well as the change in the effect size after the family was dropped (compared to the effect size from the full model; any change indicated in bold; a negative change indicating a decrease in effect size and vice versa). Families are ranked on species richness (logged number of ASVs).

| **Family** | **ASVs** | **Log (ASVs)** | **Effect size** | **l-95% CI** | **u-95% CI** | **Change in effect size** |
| --- | --- | --- | --- | --- | --- | --- |
| *Elusimicrobiaceae* | 1.000 | 0.000 | 0.012 | 0.006 | 0.018 | 0.000 |
| *Synergistaceae* | 1.000 | 0.000 | 0.012 | 0.006 | 0.018 | 0.000 |
| *Tsukamurellaceae* | 1.000 | 0.000 | 0.012 | 0.005 | 0.019 | 0.000 |
| *Rs-045* | 1.000 | 0.000 | 0.012 | 0.006 | 0.018 | 0.000 |
| *Campylobacteraceae* | 1.000 | 0.000 | 0.012 | 0.006 | 0.019 | 0.000 |
| *Barnesiellaceae* | 1.000 | 0.000 | 0.012 | 0.006 | 0.019 | 0.000 |
| *Promicromonosporaceae* | 1.000 | 0.000 | 0.012 | 0.005 | 0.018 | 0.000 |
| *Gordoniaceae* | 1.000 | 0.000 | 0.012 | 0.006 | 0.018 | 0.000 |
| *Beutenbergiaceae* | 1.000 | 0.000 | 0.012 | 0.006 | 0.019 | 0.000 |
| *Xanthobacteraceae* | 1.000 | 0.000 | 0.012 | 0.006 | 0.018 | 0.000 |
| *Rhodocyclaceae* | 1.000 | 0.000 | 0.012 | 0.006 | 0.018 | 0.000 |
| *Acholeplasmataceae* | 1.000 | 0.000 | 0.012 | 0.005 | 0.018 | 0.000 |
| *Glycomycetaceae* | 1.000 | 0.000 | 0.012 | 0.006 | 0.018 | 0.000 |
| *Dietziaceae* | 1.000 | 0.000 | 0.012 | 0.006 | 0.018 | 0.000 |
| *Succinivibrionaceae* | 1.000 | 0.000 | 0.012 | 0.005 | 0.018 | 0.000 |
| *C111* | 1.000 | 0.000 | 0.012 | 0.006 | 0.019 | 0.000 |
| *Cryptosporangiaceae* | 1.000 | 0.000 | 0.012 | 0.006 | 0.018 | 0.000 |
| *Koribacteraceae* | 1.000 | 0.000 | 0.012 | 0.006 | 0.019 | 0.000 |
| *Beijerinckiaceae* | 1.000 | 0.000 | 0.012 | 0.006 | 0.018 | 0.000 |
| *oc28* | 1.000 | 0.000 | 0.012 | 0.005 | 0.018 | 0.000 |
| *Methylophilaceae* | 1.000 | 0.000 | 0.012 | 0.006 | 0.019 | 0.000 |
| *Neisseriaceae* | 1.000 | 0.000 | 0.012 | 0.006 | 0.018 | 0.000 |
| *Cohaesibacteraceae* | 1.000 | 0.000 | 0.012 | 0.005 | 0.018 | 0.000 |
| *Nocardiopsaceae* | 1.000 | 0.000 | 0.012 | 0.005 | 0.018 | 0.000 |
| *Cerasicoccaceae* | 1.000 | 0.000 | 0.012 | 0.005 | 0.018 | 0.000 |
| *Bdellovibrionaceae* | 1.000 | 0.000 | 0.012 | 0.005 | 0.018 | 0.000 |
| *Cytophagaceae* | 1.000 | 0.000 | 0.012 | 0.005 | 0.018 | 0.000 |
| *Fimbriimonadaceae* | 1.000 | 0.000 | 0.012 | 0.005 | 0.019 | 0.000 |
| *A4b* | 1.000 | 0.000 | 0.012 | 0.005 | 0.018 | 0.000 |
| *Geodermatophilaceae* | 1.000 | 0.000 | 0.012 | 0.006 | 0.019 | 0.000 |
| *EB1017* | 1.000 | 0.000 | 0.012 | 0.006 | 0.019 | 0.000 |
| *Borreliaceae* | 1.000 | 0.000 | 0.012 | 0.006 | 0.019 | 0.000 |
| *Deinococcaceae* | 1.000 | 0.000 | 0.012 | 0.006 | 0.019 | 0.000 |
| *Armatimonadaceae* | 1.000 | 0.000 | 0.012 | 0.005 | 0.018 | 0.000 |
| *Leptospiraceae* | 1.000 | 0.000 | 0.012 | 0.006 | 0.019 | 0.000 |
| *Dolo_23* | 1.000 | 0.000 | 0.012 | 0.005 | 0.018 | 0.000 |
| *Methanosarcinaceae* | 1.000 | 0.000 | 0.012 | 0.006 | 0.019 | 0.000 |
| *Nitrososphaeraceae* | 1.000 | 0.000 | 0.012 | 0.006 | 0.018 | 0.000 |
| *Solibacteraceae* | 1.000 | 0.000 | 0.012 | 0.006 | 0.019 | 0.000 |
| *Deferribacteraceae* | 2.000 | 0.693 | 0.012 | 0.005 | 0.018 | 0.000 |
| *Paraprevotellaceae* | 2.000 | 0.693 | 0.012 | 0.006 | 0.019 | 0.000 |
| *Carnobacteriaceae* | 2.000 | 0.693 | 0.012 | 0.006 | 0.019 | 0.000 |
| *Gemellaceae* | 2.000 | 0.693 | 0.012 | 0.006 | 0.019 | 0.000 |
| *Thermaceae* | 2.000 | 0.693 | 0.012 | 0.006 | 0.018 | 0.000 |
| *Kineosporiaceae* | 2.000 | 0.693 | 0.012 | 0.006 | 0.018 | 0.000 |
| *Eubacteriaceae* | 2.000 | 0.693 | 0.012 | 0.006 | 0.019 | 0.000 |
| *Williamsiaceae* | 2.000 | 0.693 | 0.012 | 0.006 | 0.018 | 0.000 |
| *Dermacoccaceae* | 2.000 | 0.693 | 0.012 | 0.006 | 0.018 | 0.000 |
| *Ellin6075* | 2.000 | 0.693 | 0.012 | 0.006 | 0.018 | 0.000 |
| *Brevibacteriaceae* | 2.000 | 0.693 | 0.012 | 0.005 | 0.018 | 0.000 |
| *Chitinophagaceae* | 2.000 | 0.693 | 0.012 | 0.006 | 0.019 | 0.000 |
| *Acidobacteriaceae* | 2.000 | 0.693 | 0.012 | 0.006 | 0.018 | 0.000 |
| *Aurantimonadaceae* | 2.000 | 0.693 | 0.012 | 0.006 | 0.019 | 0.000 |
| *Iamiaceae* | 2.000 | 0.693 | 0.012 | 0.006 | 0.018 | 0.000 |
| *Rickettsiaceae* | 2.000 | 0.693 | 0.012 | 0.005 | 0.018 | 0.000 |
| *Planctomycetaceae* | 2.000 | 0.693 | 0.012 | 0.006 | 0.019 | 0.000 |
| *Weeksellaceae* | 2.000 | 0.693 | 0.012 | 0.005 | 0.018 | 0.000 |
| *Microthrixaceae* | 2.000 | 0.693 | 0.012 | 0.006 | 0.018 | 0.000 |
| *Prevotellaceae* | 3.000 | 1.099 | 0.012 | 0.005 | 0.018 | 0.000 |
| *Listeriaceae* | 3.000 | 1.099 | 0.012 | 0.005 | 0.019 | 0.000 |
| *Turicibacteraceae* | 3.000 | 1.099 | 0.012 | 0.006 | 0.019 | 0.000 |
| *Peptococcaceae* | 3.000 | 1.099 | 0.012 | 0.005 | 0.018 | 0.000 |
| *Sanguibacteraceae* | 3.000 | 1.099 | 0.012 | 0.006 | 0.018 | 0.000 |
| *Fusobacteriaceae* | 3.000 | 1.099 | 0.012 | 0.006 | 0.018 | 0.000 |
| *Dermabacteraceae* | 3.000 | 1.099 | 0.012 | 0.006 | 0.019 | 0.000 |
| *Sphingobacteriaceae* | 3.000 | 1.099 | 0.012 | 0.006 | 0.018 | 0.000 |
| *Flavobacteriaceae* | 3.000 | 1.099 | 0.012 | 0.006 | 0.019 | 0.000 |
| *Bifidobacteriaceae* | 4.000 | 1.386 | 0.012 | 0.006 | 0.018 | 0.000 |
| *Actinomycetaceae* | 4.000 | 1.386 | 0.012 | 0.006 | 0.018 | 0.000 |
| *Leuconostocaceae* | 4.000 | 1.386 | 0.012 | 0.005 | 0.018 | 0.000 |
| *Methylobacteriaceae* | 4.000 | 1.386 | 0.012 | 0.005 | 0.018 | 0.000 |
| *Pseudonocardiaceae* | 4.000 | 1.386 | 0.012 | 0.005 | 0.018 | 0.000 |
| *Phyllobacteriaceae* | 4.000 | 1.386 | 0.012 | 0.006 | 0.019 | 0.000 |
| *Intrasporangiaceae* | 4.000 | 1.386 | 0.012 | 0.005 | 0.018 | 0.000 |
| *Propionibacteriaceae* | 4.000 | 1.386 | 0.012 | 0.006 | 0.019 | 0.000 |
| *Rhodospirillaceae* | 4.000 | 1.386 | 0.012 | 0.005 | 0.018 | 0.000 |
| *Alcaligenaceae* | 5.000 | 1.609 | 0.012 | 0.006 | 0.019 | 0.000 |
| *Oxalobacteraceae* | 5.000 | 1.609 | 0.012 | 0.005 | 0.018 | 0.000 |
| *Peptostreptococcaceae* | 5.000 | 1.609 | 0.012 | 0.006 | 0.018 | 0.000 |
| *Corynebacteriaceae* | 5.000 | 1.609 | 0.012 | 0.006 | 0.018 | 0.000 |
| *Christensenellaceae* | 5.000 | 1.609 | 0.012 | 0.006 | 0.019 | 0.000 |
| *Methanobacteriaceae* | 5.000 | 1.609 | 0.012 | 0.005 | 0.018 | 0.000 |
| *Cellulomonadaceae* | 5.000 | 1.609 | 0.012 | 0.005 | 0.018 | 0.000 |
| *Burkholderiaceae* | 5.000 | 1.609 | 0.012 | 0.006 | 0.019 | 0.000 |
| *Spirochaetaceae* | 5.000 | 1.609 | 0.012 | 0.005 | 0.019 | 0.000 |
| *Moraxellaceae* | 5.000 | 1.609 | 0.012 | 0.005 | 0.018 | 0.000 |
| *Polyangiaceae* | 5.000 | 1.609 | 0.012 | 0.006 | 0.018 | 0.000 |
| *Legionellaceae* | 5.000 | 1.609 | 0.012 | 0.006 | 0.019 | 0.000 |
| *Gaiellaceae* | 5.000 | 1.609 | 0.012 | 0.006 | 0.019 | 0.000 |
| *Helicobacteraceae* | 6.000 | 1.792 | 0.012 | 0.006 | 0.018 | 0.000 |
| *Staphylococcaceae* | 6.000 | 1.792 | 0.012 | 0.005 | 0.018 | 0.000 |
| *Mycoplasmataceae* | 6.000 | 1.792 | 0.012 | 0.005 | 0.018 | 0.000 |
| *Verrucomicrobiaceae* | 6.000 | 1.792 | 0.012 | 0.006 | 0.019 | 0.000 |
| *Brucellaceae* | 6.000 | 1.792 | 0.012 | 0.006 | 0.019 | 0.000 |
| *Bradyrhizobiaceae* | 6.000 | 1.792 | 0.012 | 0.005 | 0.018 | 0.000 |
| *Nakamurellaceae* | 6.000 | 1.792 | 0.012 | 0.006 | 0.019 | 0.000 |
| *Micromonosporaceae* | 6.000 | 1.792 | 0.012 | 0.006 | 0.018 | 0.000 |
| *Porphyromonadaceae* | 6.000 | 1.792 | 0.012 | 0.006 | 0.018 | 0.000 |
| *Comamonadaceae* | 6.000 | 1.792 | 0.012 | 0.006 | 0.018 | 0.000 |
| *Caulobacteraceae* | 6.000 | 1.792 | 0.012 | 0.006 | 0.019 | 0.000 |
| *Odoribacteraceae* | 7.000 | 1.946 | 0.012 | 0.005 | 0.018 | 0.000 |
| *Rhodobacteraceae* | 7.000 | 1.946 | 0.012 | 0.005 | 0.018 | 0.000 |
| *Pasteurellaceae* | 7.000 | 1.946 | 0.012 | 0.006 | 0.019 | 0.000 |
| *Chthoniobacteraceae* | 7.000 | 1.946 | 0.012 | 0.005 | 0.019 | 0.000 |
| *Methylocystaceae* | 7.000 | 1.946 | 0.012 | 0.006 | 0.018 | 0.000 |
| *Sphingomonadaceae* | 7.000 | 1.946 | 0.012 | 0.006 | 0.019 | 0.000 |
| *Hyphomicrobiaceae* | 8.000 | 2.079 | 0.012 | 0.006 | 0.019 | 0.000 |
| *Thermomonosporaceae* | 8.000 | 2.079 | 0.012 | 0.006 | 0.019 | 0.000 |
| *Acetobacteraceae* | 8.000 | 2.079 | 0.012 | 0.006 | 0.018 | 0.000 |
| *Anaeroplasmataceae* | 9.000 | 2.197 | 0.012 | 0.005 | 0.018 | 0.000 |
| *Nocardiaceae* | 9.000 | 2.197 | 0.012 | 0.005 | 0.018 | 0.000 |
| *Gemmataceae* | 9.000 | 2.197 | 0.012 | 0.006 | 0.018 | 0.000 |
| *Patulibacteraceae* | 9.000 | 2.197 | 0.012 | 0.005 | 0.018 | 0.000 |
| *Conexibacteraceae* | 9.000 | 2.197 | 0.012 | 0.006 | 0.019 | 0.000 |
| *Enterococcaceae* | 10.000 | 2.303 | 0.012 | 0.006 | 0.019 | 0.000 |
| *Mycobacteriaceae* | 10.000 | 2.303 | 0.012 | 0.006 | 0.019 | 0.000 |
| *Pirellulaceae* | 10.000 | 2.303 | 0.012 | 0.006 | 0.018 | 0.000 |
| *Bacillaceae* | 12.000 | 2.485 | 0.012 | 0.005 | 0.018 | 0.000 |
| *Dehalobacteriaceae* | 13.000 | 2.565 | 0.012 | 0.005 | 0.019 | 0.000 |
| *Planococcaceae* | 13.000 | 2.565 | 0.012 | 0.006 | 0.019 | 0.000 |
| *Paenibacillaceae* | 13.000 | 2.565 | 0.012 | 0.006 | 0.019 | 0.000 |
| *Streptomycetaceae* | 13.000 | 2.565 | 0.012 | 0.006 | 0.019 | 0.000 |
| *Streptococcaceae* | 14.000 | 2.639 | 0.011 | 0.005 | 0.018 | 0.000 |
| *Coxiellaceae* | 14.000 | 2.639 | 0.012 | 0.005 | 0.018 | 0.000 |
| *Pseudomonadaceae* | 14.000 | 2.639 | 0.012 | 0.006 | 0.019 | 0.000 |
| *Micrococcaceae* | 14.000 | 2.639 | 0.012 | 0.005 | 0.018 | 0.000 |
| *Frankiaceae* | 14.000 | 2.639 | 0.012 | 0.006 | 0.019 | 0.000 |
| *Bacteroidaceae* | 16.000 | 2.773 | 0.012 | 0.005 | 0.018 | 0.000 |
| *Rhizobiaceae* | 16.000 | 2.773 | 0.012 | 0.005 | 0.018 | 0.000 |
| *Microbacteriaceae* | 17.000 | 2.833 | 0.012 | 0.006 | 0.019 | 0.000 |
| *F16* | 18.000 | 2.890 | 0.012 | 0.006 | 0.018 | 0.000 |
| *Mogibacteriaceae* | 19.000 | 2.944 | 0.012 | 0.006 | 0.018 | 0.000 |
| *Nocardioidaceae* | 21.000 | 3.045 | 0.012 | 0.006 | 0.019 | 0.000 |
| *Veillonellaceae* | 26.000 | 3.258 | 0.012 | 0.006 | 0.018 | 0.000 |
| *Erysipelotrichaceae* | 30.000 | 3.401 | 0.012 | 0.006 | 0.019 | 0.000 |
| ***Rikenellaceae*** | **31.000** | **3.434** | **0.011** | **0.004** | **0.017** | **−0.001** |
| *Desulfovibrionaceae* | 34.000 | 3.526 | 0.012 | 0.005 | 0.018 | 0.000 |
| *Clostridiaceae* | 37.000 | 3.611 | 0.012 | 0.006 | 0.018 | 0.000 |
| *Enterobacteriaceae* | 38.000 | 3.638 | 0.012 | 0.005 | 0.018 | 0.000 |
| *Isosphaeraceae* | 41.000 | 3.714 | 0.012 | 0.006 | 0.019 | 0.000 |
| ***Lactobacillaceae*** | **60.000** | **4.094** | **0.010** | **0.003** | **0.017** | **−0.001** |
| *Coriobacteriaceae* | 63.000 | 4.143 | 0.012 | 0.006 | 0.019 | 0.000 |
| ***Muribaculaceae*** | **206.000** | **5.328** | **0.007** | **0.001** | **0.014** | **−0.005** |
| *Ruminococcaceae* | 396.000 | 5.981 | 0.014 | 0.007 | 0.020 | 0.002 |
| *Lachnospiraceae* | 756.000 | 6.628 | 0.016 | 0.009 | 0.022 | 0.004 |
| Unknown | 901.000 | 6.804 | 0.015 | 0.009 | 0.022 | 0.003 |
