# Supplementary material for: Maternal transmission gives way to social transmission during gut microbiota assembly in wild mice
Source: Anim Microbiome. 2023 May 31;5:29. doi: 10.1186/s42523-023-00247-7 (PMC10230743; doi:10.1186/s42523-023-00247-7)
Supplement: Supplementary file 4 — Additional file 4: Table S4. Results of 146 brms models testing the interaction between mother-offspring status and age class, in each of which a single bacterial family was dropped. For each dropped family, we include the species richness of the dropped family, the effect size and its 95% credible interval after the family was dropped, as well as the change in the effect size after the family was dropped (compared to the effect size from the full model; any change indicated in bold; a negative change indicating a decrease in effect size and vice versa). Families are ranked on species richness (logged number of ASVs) [file 42523_2023_247_MOESM4_ESM.docx]

**Table S4** Results of 146 *brms* models testing the interaction between mother-offspring status and age class, in each of which a single bacterial family was dropped. For each dropped family, we include the species richness of the dropped family, the effect size and its 95% credible interval after the family was dropped, as well as the change in the effect size after the family was dropped (compared to the effect size from the full model; any change indicated in bold; a negative change indicating a decrease in effect size and vice versa). Families are ranked on species richness (logged number of ASVs).

| **Family** | **ASVs** | **Log (ASVs)** | **Effect size** | **l−95% CI** | **u−95% CI** | **Change in effect size** |
| --- | --- | --- | --- | --- | --- | --- |
| *Elusimicrobiaceae* | 1.000 | 0.000 | −0.016 | −0.026 | −0.006 | 0.000 |
| *Synergistaceae* | 1.000 | 0.000 | −0.015 | −0.026 | −0.005 | 0.000 |
| *Tsukamurellaceae* | 1.000 | 0.000 | −0.015 | −0.026 | −0.005 | 0.000 |
| *Rs−045* | 1.000 | 0.000 | −0.015 | −0.025 | −0.004 | 0.000 |
| *Campylobacteraceae* | 1.000 | 0.000 | −0.015 | −0.025 | −0.005 | 0.000 |
| *Barnesiellaceae* | 1.000 | 0.000 | −0.015 | −0.026 | −0.006 | 0.000 |
| *Promicromonosporaceae* | 1.000 | 0.000 | −0.015 | −0.025 | −0.005 | 0.000 |
| *Gordoniaceae* | 1.000 | 0.000 | −0.015 | −0.026 | −0.005 | 0.000 |
| *Beutenbergiaceae* | 1.000 | 0.000 | −0.015 | −0.025 | −0.005 | 0.000 |
| *Xanthobacteraceae* | 1.000 | 0.000 | −0.015 | −0.026 | −0.005 | 0.000 |
| *Rhodocyclaceae* | 1.000 | 0.000 | −0.015 | −0.026 | −0.005 | 0.000 |
| *Acholeplasmataceae* | 1.000 | 0.000 | −0.015 | −0.025 | −0.004 | 0.000 |
| *Glycomycetaceae* | 1.000 | 0.000 | −0.015 | −0.025 | −0.004 | 0.000 |
| *Dietziaceae* | 1.000 | 0.000 | −0.015 | −0.025 | −0.004 | 0.000 |
| *Succinivibrionaceae* | 1.000 | 0.000 | −0.015 | −0.026 | −0.005 | 0.000 |
| *C111* | 1.000 | 0.000 | −0.015 | −0.026 | −0.005 | 0.000 |
| *Cryptosporangiaceae* | 1.000 | 0.000 | −0.015 | −0.025 | −0.005 | 0.000 |
| *Koribacteraceae* | 1.000 | 0.000 | −0.015 | −0.025 | −0.004 | 0.000 |
| *Beijerinckiaceae* | 1.000 | 0.000 | −0.015 | −0.025 | −0.005 | 0.000 |
| *oc28* | 1.000 | 0.000 | −0.015 | −0.027 | −0.006 | 0.000 |
| *Methylophilaceae* | 1.000 | 0.000 | −0.015 | −0.026 | −0.005 | 0.000 |
| *Neisseriaceae* | 1.000 | 0.000 | −0.015 | −0.025 | −0.005 | 0.000 |
| *Cohaesibacteraceae* | 1.000 | 0.000 | −0.015 | −0.025 | −0.005 | 0.000 |
| *Nocardiopsaceae* | 1.000 | 0.000 | −0.015 | −0.025 | −0.004 | 0.000 |
| *Cerasicoccaceae* | 1.000 | 0.000 | −0.015 | −0.025 | −0.005 | 0.000 |
| *Bdellovibrionaceae* | 1.000 | 0.000 | −0.015 | −0.025 | −0.005 | 0.000 |
| *Cytophagaceae* | 1.000 | 0.000 | −0.015 | −0.026 | −0.005 | 0.000 |
| *Fimbriimonadaceae* | 1.000 | 0.000 | −0.015 | −0.025 | −0.004 | 0.000 |
| *A4b* | 1.000 | 0.000 | −0.015 | −0.026 | −0.004 | 0.000 |
| *Geodermatophilaceae* | 1.000 | 0.000 | −0.015 | −0.027 | −0.006 | 0.000 |
| *EB1017* | 1.000 | 0.000 | −0.015 | −0.025 | −0.005 | 0.000 |
| *Borreliaceae* | 1.000 | 0.000 | −0.015 | −0.026 | −0.005 | 0.000 |
| *Deinococcaceae* | 1.000 | 0.000 | −0.015 | −0.026 | −0.006 | 0.000 |
| *Armatimonadaceae* | 1.000 | 0.000 | −0.015 | −0.026 | −0.005 | 0.000 |
| *Leptospiraceae* | 1.000 | 0.000 | −0.015 | −0.026 | −0.006 | 0.000 |
| *Dolo_23* | 1.000 | 0.000 | −0.015 | −0.026 | −0.005 | 0.000 |
| *Methanosarcinaceae* | 1.000 | 0.000 | −0.015 | −0.025 | −0.005 | 0.000 |
| *Nitrososphaeraceae* | 1.000 | 0.000 | −0.015 | −0.025 | −0.005 | 0.000 |
| *Solibacteraceae* | 1.000 | 0.000 | −0.015 | −0.026 | −0.005 | 0.000 |
| *Deferribacteraceae* | 2.000 | 0.693 | −0.015 | −0.027 | −0.005 | 0.000 |
| *Paraprevotellaceae* | 2.000 | 0.693 | −0.015 | −0.025 | −0.005 | 0.000 |
| *Carnobacteriaceae* | 2.000 | 0.693 | −0.015 | −0.027 | −0.006 | 0.000 |
| *Gemellaceae* | 2.000 | 0.693 | −0.015 | −0.026 | −0.005 | 0.000 |
| *Thermaceae* | 2.000 | 0.693 | −0.015 | −0.026 | −0.005 | 0.000 |
| *Kineosporiaceae* | 2.000 | 0.693 | −0.015 | −0.026 | −0.006 | 0.000 |
| *Eubacteriaceae* | 2.000 | 0.693 | −0.015 | −0.025 | −0.005 | 0.000 |
| *Williamsiaceae* | 2.000 | 0.693 | −0.015 | −0.026 | −0.006 | 0.000 |
| *Dermacoccaceae* | 2.000 | 0.693 | −0.015 | −0.026 | −0.006 | 0.000 |
| *Ellin6075* | 2.000 | 0.693 | −0.015 | −0.026 | −0.005 | 0.000 |
| *Brevibacteriaceae* | 2.000 | 0.693 | −0.015 | −0.026 | −0.004 | 0.000 |
| *Chitinophagaceae* | 2.000 | 0.693 | −0.015 | −0.026 | −0.005 | 0.000 |
| *Acidobacteriaceae* | 2.000 | 0.693 | −0.015 | −0.025 | −0.005 | 0.000 |
| *Aurantimonadaceae* | 2.000 | 0.693 | −0.015 | −0.025 | −0.005 | 0.000 |
| *Iamiaceae* | 2.000 | 0.693 | −0.015 | −0.025 | −0.005 | 0.000 |
| *Rickettsiaceae* | 2.000 | 0.693 | −0.015 | −0.026 | −0.005 | 0.000 |
| *Planctomycetaceae* | 2.000 | 0.693 | −0.015 | −0.025 | −0.005 | 0.000 |
| *Weeksellaceae* | 2.000 | 0.693 | −0.015 | −0.025 | −0.004 | 0.000 |
| *Microthrixaceae* | 2.000 | 0.693 | −0.015 | −0.026 | −0.006 | 0.000 |
| *Prevotellaceae* | 3.000 | 1.099 | −0.015 | −0.026 | −0.005 | 0.000 |
| *Listeriaceae* | 3.000 | 1.099 | −0.015 | −0.027 | −0.005 | 0.000 |
| *Turicibacteraceae* | 3.000 | 1.099 | −0.015 | −0.027 | −0.005 | 0.000 |
| *Peptococcaceae* | 3.000 | 1.099 | −0.015 | −0.026 | −0.006 | 0.000 |
| *Sanguibacteraceae* | 3.000 | 1.099 | −0.015 | −0.025 | −0.005 | 0.000 |
| *Fusobacteriaceae* | 3.000 | 1.099 | −0.015 | −0.026 | −0.005 | 0.000 |
| *Dermabacteraceae* | 3.000 | 1.099 | −0.015 | −0.025 | −0.004 | 0.000 |
| *Sphingobacteriaceae* | 3.000 | 1.099 | −0.015 | −0.026 | −0.005 | 0.000 |
| *Flavobacteriaceae* | 3.000 | 1.099 | −0.015 | −0.026 | −0.005 | 0.000 |
| *Bifidobacteriaceae* | 4.000 | 1.386 | −0.015 | −0.026 | −0.005 | 0.000 |
| *Actinomycetaceae* | 4.000 | 1.386 | −0.015 | −0.027 | −0.005 | 0.000 |
| *Leuconostocaceae* | 4.000 | 1.386 | −0.015 | −0.025 | −0.005 | 0.000 |
| *Methylobacteriaceae* | 4.000 | 1.386 | −0.015 | −0.025 | −0.005 | 0.000 |
| *Pseudonocardiaceae* | 4.000 | 1.386 | −0.015 | −0.025 | −0.005 | 0.000 |
| *Phyllobacteriaceae* | 4.000 | 1.386 | −0.015 | −0.025 | −0.005 | 0.000 |
| *Intrasporangiaceae* | 4.000 | 1.386 | −0.015 | −0.026 | −0.006 | 0.000 |
| *Propionibacteriaceae* | 4.000 | 1.386 | −0.015 | −0.026 | −0.005 | 0.000 |
| *Rhodospirillaceae* | 4.000 | 1.386 | −0.015 | −0.026 | −0.005 | 0.000 |
| *Alcaligenaceae* | 5.000 | 1.609 | −0.016 | −0.026 | −0.005 | 0.000 |
| *Oxalobacteraceae* | 5.000 | 1.609 | −0.015 | −0.025 | −0.004 | 0.000 |
| *Peptostreptococcaceae* | 5.000 | 1.609 | −0.015 | −0.026 | −0.005 | 0.000 |
| *Corynebacteriaceae* | 5.000 | 1.609 | −0.016 | −0.026 | −0.006 | 0.000 |
| *Christensenellaceae* | 5.000 | 1.609 | −0.015 | −0.027 | −0.006 | 0.000 |
| *Methanobacteriaceae* | 5.000 | 1.609 | −0.015 | −0.025 | −0.005 | 0.000 |
| *Cellulomonadaceae* | 5.000 | 1.609 | −0.015 | −0.025 | −0.005 | 0.000 |
| *Burkholderiaceae* | 5.000 | 1.609 | −0.015 | −0.025 | −0.005 | 0.000 |
| *Spirochaetaceae* | 5.000 | 1.609 | −0.015 | −0.025 | −0.005 | 0.000 |
| *Moraxellaceae* | 5.000 | 1.609 | −0.015 | −0.026 | −0.005 | 0.000 |
| *Polyangiaceae* | 5.000 | 1.609 | −0.015 | −0.025 | −0.006 | 0.000 |
| *Legionellaceae* | 5.000 | 1.609 | −0.015 | −0.025 | −0.005 | 0.000 |
| *Gaiellaceae* | 5.000 | 1.609 | −0.015 | −0.025 | −0.005 | 0.000 |
| ***Helicobacteraceae*** | **6.000** | **1.792** | **−0.014** | **−0.024** | **−0.003** | **−0.002** |
| *Staphylococcaceae* | 6.000 | 1.792 | −0.015 | −0.027 | −0.005 | 0.000 |
| *Mycoplasmataceae* | 6.000 | 1.792 | −0.015 | −0.025 | −0.005 | 0.000 |
| *Verrucomicrobiaceae* | 6.000 | 1.792 | −0.016 | −0.025 | −0.005 | 0.000 |
| *Brucellaceae* | 6.000 | 1.792 | −0.015 | −0.025 | −0.004 | 0.000 |
| *Bradyrhizobiaceae* | 6.000 | 1.792 | −0.015 | −0.025 | −0.005 | 0.000 |
| *Nakamurellaceae* | 6.000 | 1.792 | −0.015 | −0.026 | −0.004 | 0.000 |
| *Micromonosporaceae* | 6.000 | 1.792 | −0.015 | −0.027 | −0.006 | 0.000 |
| *Porphyromonadaceae* | 6.000 | 1.792 | −0.015 | −0.027 | −0.006 | 0.000 |
| *Comamonadaceae* | 6.000 | 1.792 | −0.016 | −0.026 | −0.005 | 0.000 |
| *Caulobacteraceae* | 6.000 | 1.792 | −0.015 | −0.026 | −0.006 | 0.000 |
| ***Odoribacteraceae*** | **7.000** | **1.946** | **−0.014** | **−0.025** | **−0.004** | **−0.001** |
| *Rhodobacteraceae* | 7.000 | 1.946 | −0.015 | −0.026 | −0.006 | 0.000 |
| *Pasteurellaceae* | 7.000 | 1.946 | −0.015 | −0.025 | −0.005 | 0.000 |
| *Chthoniobacteraceae* | 7.000 | 1.946 | −0.015 | −0.025 | −0.005 | 0.000 |
| *Methylocystaceae* | 7.000 | 1.946 | −0.015 | −0.026 | −0.006 | 0.000 |
| *Sphingomonadaceae* | 7.000 | 1.946 | −0.015 | −0.026 | −0.005 | 0.000 |
| *Hyphomicrobiaceae* | 8.000 | 2.079 | −0.015 | −0.026 | −0.005 | 0.000 |
| *Thermomonosporaceae* | 8.000 | 2.079 | −0.015 | −0.025 | −0.004 | 0.000 |
| *Acetobacteraceae* | 8.000 | 2.079 | −0.015 | −0.026 | −0.006 | 0.000 |
| *Anaeroplasmataceae* | 9.000 | 2.197 | −0.016 | −0.026 | −0.005 | 0.000 |
| *Nocardiaceae* | 9.000 | 2.197 | −0.015 | −0.025 | −0.005 | 0.000 |
| *Gemmataceae* | 9.000 | 2.197 | −0.015 | −0.026 | −0.005 | 0.000 |
| *Patulibacteraceae* | 9.000 | 2.197 | −0.015 | −0.026 | −0.006 | 0.000 |
| *Conexibacteraceae* | 9.000 | 2.197 | −0.015 | −0.025 | −0.005 | 0.000 |
| *Enterococcaceae* | 10.000 | 2.303 | −0.016 | −0.026 | −0.006 | 0.000 |
| *Mycobacteriaceae* | 10.000 | 2.303 | −0.015 | −0.026 | −0.006 | 0.000 |
| *Pirellulaceae* | 10.000 | 2.303 | −0.016 | −0.025 | −0.005 | 0.000 |
| *Bacillaceae* | 12.000 | 2.485 | −0.016 | −0.026 | −0.005 | 0.000 |
| *Dehalobacteriaceae* | 13.000 | 2.565 | −0.015 | −0.026 | −0.006 | 0.000 |
| *Planococcaceae* | 13.000 | 2.565 | −0.015 | −0.026 | −0.006 | 0.000 |
| *Paenibacillaceae* | 13.000 | 2.565 | −0.015 | −0.025 | −0.004 | 0.000 |
| *Streptomycetaceae* | 13.000 | 2.565 | −0.015 | −0.026 | −0.005 | 0.000 |
| ***Streptococcaceae*** | **14.000** | **2.639** | **−0.014** | **−0.025** | **−0.005** | **−0.001** |
| *Coxiellaceae* | 14.000 | 2.639 | −0.015 | −0.026 | −0.005 | 0.000 |
| *Pseudomonadaceae* | 14.000 | 2.639 | −0.016 | −0.026 | −0.005 | 0.000 |
| *Micrococcaceae* | 14.000 | 2.639 | −0.015 | −0.025 | −0.005 | 0.000 |
| *Frankiaceae* | 14.000 | 2.639 | −0.016 | −0.026 | −0.006 | 0.000 |
| ***Bacteroidaceae*** | **16.000** | **2.773** | **−0.015** | **−0.025** | **−0.004** | **−0.001** |
| *Rhizobiaceae* | 16.000 | 2.773 | −0.015 | −0.026 | −0.005 | 0.000 |
| *Microbacteriaceae* | 17.000 | 2.833 | −0.015 | −0.026 | −0.005 | 0.000 |
| *F16* | 18.000 | 2.890 | −0.015 | −0.026 | −0.005 | 0.000 |
| *Mogibacteriaceae* | 19.000 | 2.944 | −0.016 | −0.027 | −0.006 | 0.000 |
| *Nocardioidaceae* | 21.000 | 3.045 | −0.015 | −0.026 | −0.004 | 0.000 |
| *Veillonellaceae* | 26.000 | 3.258 | −0.015 | −0.025 | −0.004 | 0.000 |
| *Erysipelotrichaceae* | 30.000 | 3.401 | −0.015 | −0.025 | −0.005 | 0.000 |
| *Rikenellaceae* | 31.000 | 3.434 | −0.016 | −0.026 | −0.005 | 0.000 |
| ***Desulfovibrionaceae*** | **34.000** | **3.526** | **−0.015** | **−0.025** | **−0.005** | **−0.001** |
| *Clostridiaceae* | 37.000 | 3.611 | −0.015 | −0.025 | −0.005 | 0.000 |
| *Enterobacteriaceae* | 38.000 | 3.638 | −0.015 | −0.025 | −0.005 | 0.000 |
| *Isosphaeraceae* | 41.000 | 3.714 | −0.015 | −0.025 | −0.005 | 0.000 |
| *Lactobacillaceae* | 60.000 | 4.094 | −0.015 | −0.025 | −0.005 | 0.000 |
| ***Coriobacteriaceae*** | **63.000** | **4.143** | **−0.015** | **−0.026** | **−0.005** | **−0.001** |
| ***Muribaculaceae*** | **206.000** | **5.328** | **−0.009** | **−0.019** | **0.001** | **−0.007** |
| *Ruminococcaceae* | 396.000 | 5.981 | −0.017 | −0.028 | −0.008 | 0.002 |
| *Lachnospiraceae* | 756.000 | 6.628 | −0.021 | −0.032 | −0.011 | 0.006 |
| Unknown | 901.000 | 6.804 | −0.021 | −0.032 | −0.010 | 0.005 |
